# Metabolic engineering of CHO cells for the development of a robust protein production platform

**DOI:** 10.1371/journal.pone.0181455

**Published:** 2017-08-01

**Authors:** Sanjeev Kumar Gupta, Santosh K. Srivastava, Ankit Sharma, Vaibhav H. H. Nalage, Darshita Salvi, Hiralal Kushwaha, Nikhil B. Chitnis, Pratyoosh Shukla

**Affiliations:** 1 Advanced Biotech Lab, Ipca Laboratories Ltd., Plot#125, Kandivli Industrial Estate, Kandivli (west), Mumbai, Maharashtra, India; 2 Enzyme Technology and Protein Bioinformatics Laboratory, Department of Microbiology, Maharshi Dayanand University, Rohtak, Haryana-India; University of Nebraska Medical Center, UNITED STATES

## Abstract

Chinese hamster ovary (CHO) cells are the most preferred mammalian host used for the bio-pharmaceutical production. A major challenge in metabolic engineering is to balance the flux of the tuned heterogonous metabolic pathway and achieve efficient metabolic response in a mammalian cellular system. Pyruvate carboxylase is an important network element for the cytoplasmic and mitochondrial metabolic pathway and efficiently contributes in enhancing the energy metabolism. The lactate accumulation in cell culture can be reduced by re-wiring of the pyruvate flux in engineered cells. In the present work, we over-expressed the yeast cytosolic pyruvate carboxylase (PYC2) enzyme in CHO cells to augment pyruvate flux towards the TCA cycle. The dual selection strategy is adopted for the screening and isolation of CHO clones containing varying number of PYC2 gene load and studied their cellular kinetics. The enhanced PYC2 expression has led to enhanced pyruvate flux which, thus, allowed reduced lactate accumulation up to 4 folds and significant increase in the cell density and culture longevity. With this result, engineered cells have shown a significant enhanced antibody expression up to 70% with improved product quality (~3 fold) as compared to the parental cells. The PYC2 engineering allowed overall improved cell performance with various advantages over parent cells in terms of pyruvate, glucose, lactate and cellular energy metabolism. This study provides a potential expression platform for a bio-therapeutic protein production in a controlled culture environment.

## Introduction

The bio-pharmaceutical market is significantly growing worldwide due to increasing prevalence of chronic diseases, rising aging population and technological advancements in bio-pharmaceuticals [1–4]. Global biopharmaceuticals market was valued at $162 bn in 2014 and is expected to reach an estimated value of $278 bn by 2020 [5,6]. In most of the bio-pharmaceutical industries, the Chinese hamster ovary (CHO) cells are predominantly used as an expression host for the production of recombinant monoclonal antibodies [1,2,7,8]. Majority of the bio-pharmaceutical drugs are produced by CHO cells using a fed batch cell culture process, since a fed-batch process has greatly helped in enhancing the production yield in cell culture broth [9]. In a fed-batch upstream process, usually the unwanted waste products such as lactic acid and ammonia are accumulated over a period of time, which can hamper the cell growth as well as product quality attributes of a recombinant product [10]. The CHO based fed-batch cell culture process leads to high lactate accumulation due to uncontrolled glucose metabolism which can cause medium acidification and osmolality changes as a result of alkali addition done to control the culture pH [11–13]. CHO cells producing therapeutic proteins require constant availability of carbon, nitrogen, energy (ATP) and reductant (NADPH) to sustain their anabolic functions [14]. The main stream of carbon metabolism consist of three major metabolic pathways, glycolysis, pentose phosphate pathways (PPP) and tricarboxylic acid (TCA) cycle [15]. Glycolysis is the main catabolic pathways through which glucose is oxidized and at the end, one molecule of glucose is converted into two molecules of pyruvate which ultimately enters to the mitochondria and oxidized in the TCA cycle. Cellular metabolism of the CHO cells in a fed-batch mode require a high rate of glycolysis, consequentially triggers the accumulation of pyruvate. Due to poor connectivity of the mitochondrial and cytosolic metabolic system, most of the accumulated pyruvate flux drive the production of the lactate by lactate dehydrogenase enzyme (LDH) [16,17]. Till date more than a few approaches have been attempted for the cell culture waste reduction using either metabolic engineering of a production host or process engineering of the cell culture process. The cell culture process strategies includes the substitution of nutrients, for example, glucose with galactose or pyruvate or substituting glutamine with asparagine or glutamate [18–20]. Although, the substitution of nutrients allows reduced accumulation of waste products, however often results into compromised cell growth [21]. The tuning of the enzymes involved in metabolic pathway of a cellular system is critical for reducing metabolic burden and decreasing accumulation of toxic intermediates without affecting the health and viability of an engineered host [21–26]. In previous studies various metabolic engineering approaches have also been investigated to generate cell lines with improved metabolic characteristics [22–30]. To date, various attempts have been made for the reduction of lactate accumulation by expression of the cytosolic yeast pyruvate carboxylase 2 (PYC2) gene in mammalian cells BHK and HEK293 cell which allowed either increased protein production or improved cell growth [28–30]. In another case, PYC2 was expressed in the CHO cells, which allowed decreased specific lactate production [22,31,32]. Recently, Wilkens and Gerdtzen (2015) demonstrated that over-expression of PYC2 in CHO cells allowed prolonged cell culture and reduced lactate/glucose ratio by 25%, but, the specific productivity as reduced by 50% thus affected the overall volumetric antibody expression.

Whereas in another recent work, the antibody volumetric productivity shown to be increased and specific productivity marginally reduced due to improved cell efficiency caused by over expression of PYC2 in an antibody producing CHO cells [33].

In the current study, we adopted a yeast derived gene encoding for enzyme pyruvate carboxylase and the codon of which was optimized for CHO cells to increase the translational machinery and higher PYC2 expression in the CHO cells. We also explored the role of PYC2 gene expression in mammalian cellular system by generating a stable CHO cell pool containing a diverse population PYC2 gene load in their genome.

Further, we screened the PYC2 engineered CHO clones from the heterogenous population of PYC2 engineered cell pool. Subsequently, the single cell clone from the panel of PYC2 clones were selected based on the culture performance and their metabolic profile. In addition, the kinetics of an engineered cell in different culture conditions is also studied in comparison with the parental cells. Based on various biochemical and metabolic analysis, we established a metabolically controlled CHO platform having enhanced PYC2 gene load and able to sustain in a fed-batch process for extended period of time with very high cell density, high glucose consumption rate, and significant reduced lactate accumulation. The PYC2 engineered cells were also tested for the expression of a therapeutic monoclonal antibody which has shown a significant improved culture performance thus enhanced product titer and quality attributes [34–36]. There is no report published so far which describes the impact of PYC2 gene load on CHO cells performance as well as effect of enhanced expression on glycoforms of a chimeric monoclonal antibody [37–40]. We also investigated the impact of the PYC2 gene load on N-glycosylation pattern of a therapeutic mAb and its impact on the amino acid and, cell energy metabolism in terms of NAD+/NADH ratio, N-glycosylation pattern as well as charge variant distribution.

## Results

### Selection of metabolically optimized transfected CHO cell pools

To enhance the cellular metabolism and improve the cytosolic and mitochondrial metabolic network **(S1 Fig)**, we engineered the CHO cellular system by increasing the PYC2 gene load in the CHO cell pool. In this study, we used codon optimized (for CHO) yeast pyruvate carboxylase (PYC2) gene which is expressed in the cytosol of the CHO cells. The PYC2 play a key role in controlling the Pyruvate accumulation by driving the cytosolic pyruvate towards the kreb’s cycle of the metabolic pathway”. Increased gene copies expressing a metabolically optimized PYC2 gene triggers the pyruvate flux towards the mitochondrial metabolic network efficiently and connects the glycolysis to the TCA cycle for an enhanced internal cellular energy metabolism.

**Fig 1:**
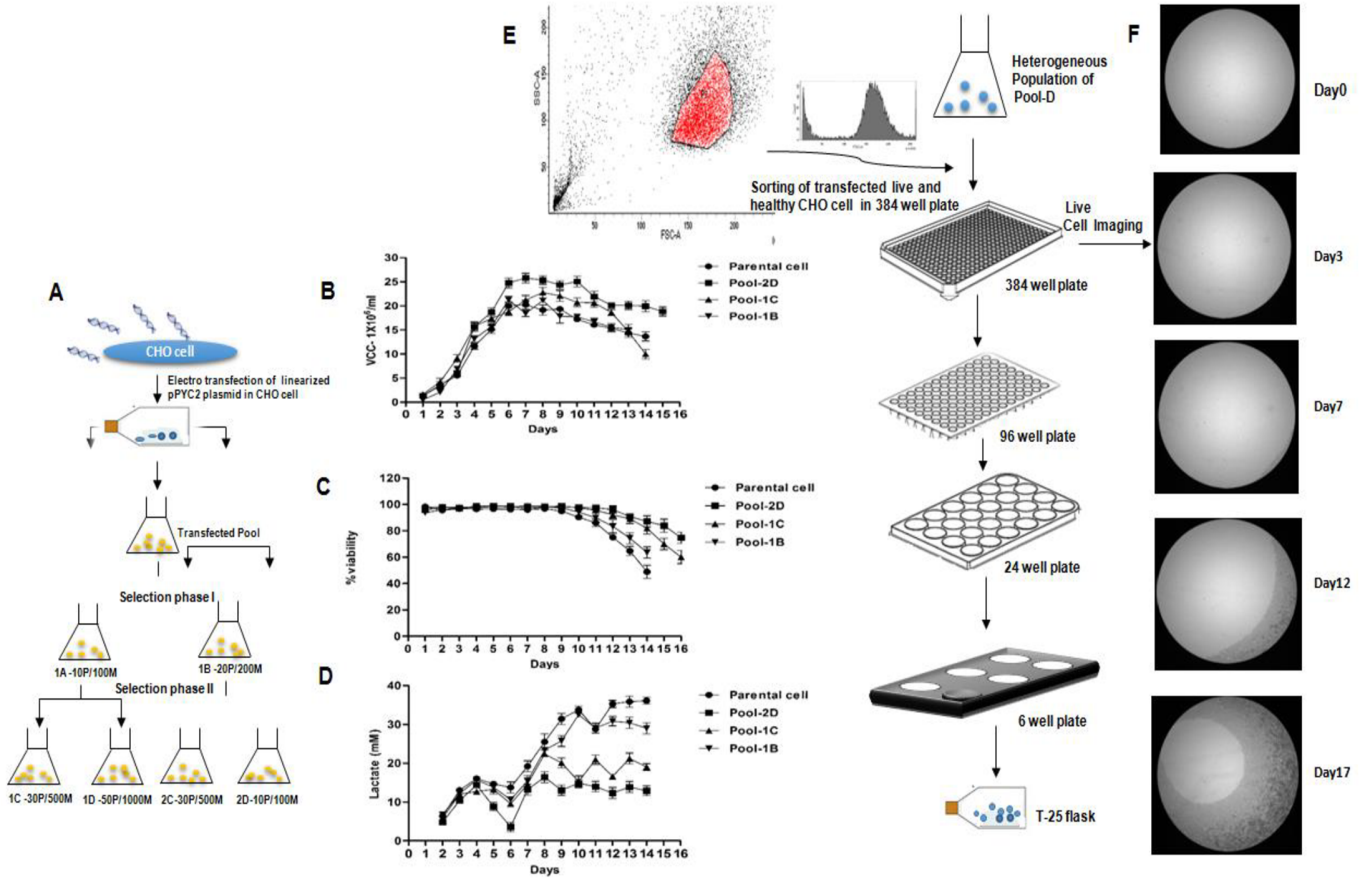
Cell pool generation and cell line development for PYC2 engineering. **(A-D)** Schematic diagram of two-phase selection of pMPYC transfected CHO cells with varying concentration of MTX and puromycin. (**B-D)** Cell density, viability and lactate production profiles for stable pool 1B, pool 1C and pool 2D in fed-batch culture mode as a function of time. **(E-F**) Flow diagram depicts the steps followed to obtain a clonal CHO cell line from the heterogeneous population of a stable pool. Flow-cytometry used to gate the healthy cells population and sort the single CHO cell in a 384 well plate. Live cell imager is used to monitor the clonality and growth of the colony/cell in the consecutive days. Single clones are expanded from 384 well plates to T25 flask.

The CHO-S cells (suspension) were transfected with the plasmid pMPYC bearing PYC2 gene by using neon electroporatorion and stable cell pools were generated. From the heterogeneous transfected pool, it was challenging task to select an engineered clone with increased gene copies expressing a metabolically optimized PYC2 gene, which triggers the pyruvate flux towards the mitochondrial metabolic network and efficiently connect the glycolysis to the TCA cycle for an enhanced internal cellular energy generation. To fish-out a desired CHO clone from the transfected pools, we applied two-phase selection scheme, wherein we generated the transfected CHO cell population using varying concentration of selection agents’ puromycin and MTX **(Fig 1A)**. The gradual increase of the selection pressure from low to high concentration allowed stringent selection of the desired cell pool by eliminating poor cells/clones derived from highly heterogeneous population. Selected pools had a mix population of transfected PYC2-CHO cells harboring the varying PYC2 gene copies and also changing PYC2 expression pattern due to the random integration event as well as position effect in the CHO genome. To assess the metabolic performances of each pool, we, further chose pool 1B, 1C and 2D for the evaluation of PYC2 expression efficiency and subsequently performed a fed-batch shake flask experiment using CD-Forti-CHO basal medium and Acti-feed A and B (GE, USA) feed supplements. We assessed the metabolic profile (lactate and glucose), cell viability and growth pattern of these pools from exponential to the stationary phase of the cell cycle and compared few important parameters with the parental CHO cells (non-transfected). We observed that, the pool 1B (selected from Phase I, with 200nM MTX and 20ug/ml Puromycin), pool 1C (selected from phase II, with 500nM MTX and 30ug/ml Puromycin) and pool 2D (selected from phase II, with 1000nM MTX and 50ug/ml Puromycin) have shown a significant difference among themselves as well as with the parental CHO cells in terms of cell viability and cell density profile. In pool 1B, the peak cell density achieved was up to 20 million cells/mL, however, the cell viability started declining from day 10 onwards and reached up to approximately 72% on day 14, whereas Pool 1C shown cell density up to 23 million cells/mL and viability started declining from day 11 onwards and reached up to approximately 83% on the same day (day 14). In contrast, Pool 2D selected from highest concentration of selection agents, showed significant extended cell viability as well as highest cell density amongst the pools **(Fig 1B and 1C)**. In pool 2D, the peak cell density achieved was around 26 million cells/mL and cell viability on day 14 was approximately 92% which is 10–20% higher than other two pools including parental CHO cells **(Fig 1B and 1C)** as described above. Furthermore, we analyzed the metabolic behavior of the cells in terms of lactate accumulation and consumption rates, and found that pool 2D showed a significant difference in the lactate metabolism as compared to rest of the pools and parental cell. The lactate profile of pool 2D and 1C and Pool 1B and Parental CHO cells were similar to each other, however, pool 2D has shown significant lower lactate production **(Fig 1D)** compared to other cell pools. Since, these pools have heterogeneous cell population harboring varying PYC2 gene load and during fed-batch culture the cumulative effect of heterogeneous population showed a variation in the cell viability, density and lactate profile, we, therefore selected pool 2D as a final pool for subsequent single cell cloning, screening and establishing a potential engineered cell candidate. The single cell sorting was performed using FACS-Aria III (BD bioscience, USA) by setting up an efficient gating for live and good margin/edge cell shape **(Fig 1E).** The cell sorting was performed under the white background without using any labeled agent/antibody. The sorted single cell initially grown in 384 well plate and further their growth patterns was monitored by using live cell imager (Thermo Fischer Scientific, USA) at different time intervals starting from the day zero to day 17 **(Fig 1F)**. Based on the morphology, growth and clonality of the cells/clones, we picked up those colonies which were derived from a single cell only (confirmed by cell imager) and the selected colonies were then transferred and scaled-up gradually from low to high volume into the multi well plates followed by T-25 flask as described in materials and methods **(Fig 1E and 1F)**. The clone stability study of PYC2 clone #12 was also performed till 70 generations in a basal media CD-Forti-CHO supplemented with 6mM glutamine (Shake flask) with and without selection pressure and was found stable up to 50 generations, which is more than sufficient for further studies.

### Comparative fed- batch study of the selected clones

**Fig 2:**
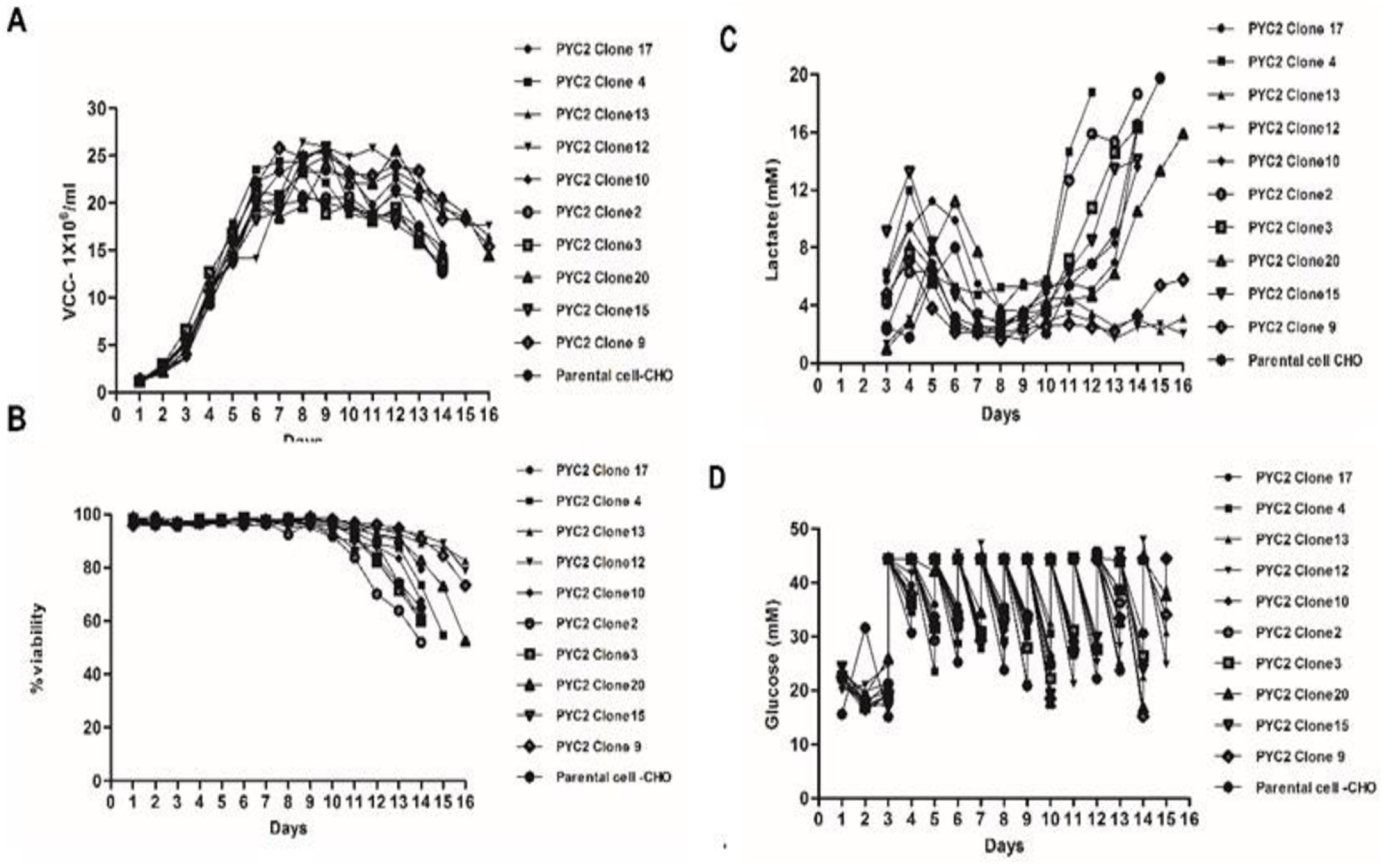
A comparative shake flask fed-batch study of the screened clones expressing PYC2 gene. **Graph representing culture profile: (A)** Cell density (**B)** cell viability (**C)** lactate profile (**D)** glucose consumption profile for PYC2-expressing cells in shake-flask culture.

In this part of the study, we performed a shake flask fed-batch experiment with multiple selected single cell clones derived from the pool 2D. In previous study performed in-house, it has been observed that the CHO cells producing recombinant antibody in CD-Forti-CHO medium and Acti-feed A and B supplement triggers high lactate production during the exponential and stationary phase of the cell culture which leads to a potential drop in the cell viability, thus early batch termination with poor protein expression occurs. To address above issue, we assessed PYC2 expression in randomly selected single cell clones by mRNA RT-PCR study. Furthermore, based on the relative mRNA expression profile of the integrated PYC2 gene, we selected top 10 clones for the further expression and metabolic studies. Top 10 selected clones, were cultivated in a complete CD-Forti-CHO medium and Acti-feed A and B feeds in a shake flask fed-batch culture using standard cell culture process parameters (described in materials and methods). In shake flask fed-batch study, we supplemented the growing culture with 6mM glutamine and 8g/L (44.5mM) glucose, and fed the culture with 1.3% Acti-feed A and 0.13% Acti-feed B. We measured the cell concentration, glucose, lactate, cell density and viability throughout the shake flask study. In the fed batch mode, clone#12 and clone#13 showed significant increased cell density with sustained stationary phase before moving towards decline phase of the cell growth **(Fig 2A)**. The cell viability of PYC2 expressing clone #12 and clone #13 were 94% and 90%, respectively till day 14 and maximum cell density achieved was up to 26 million cells/mL and 25.3 million cells/mL, respectively **(Fig 2A and 2B)**. In addition, these clones showed a good lactate consumption profile (< 4mM) as compared to other screened clones (up to 5 folds difference in lactate consumption profile) **(Fig 2C)**. Further, we observed that the clone# 4, 10 and 20 also exhibited the cell density around 24 million cells/mL, but, were not able to sustain for an extended period as observed in the case of clone #12 and clone #13 **(Fig 2A and 2B)**. The viability of these clones started declining after day 10 and dropped down in the range of 70 to 80% by day 14. Interestingly, these clones produced lactic acid rapidly when culture shifted from late exponential phase to the early stationary phase. Distinctive metabolic behavior of the PYC2 clones in the fed-batch culture indicated that, each clone differs in their metabolic activity and carbon utilization rate. This observation clearly indicated that the PYC2 gene load might have changed the pyruvate flux as well as overall metabolic networking of the cytoplasm and mitochondrial matrix.

**Fig 3:**
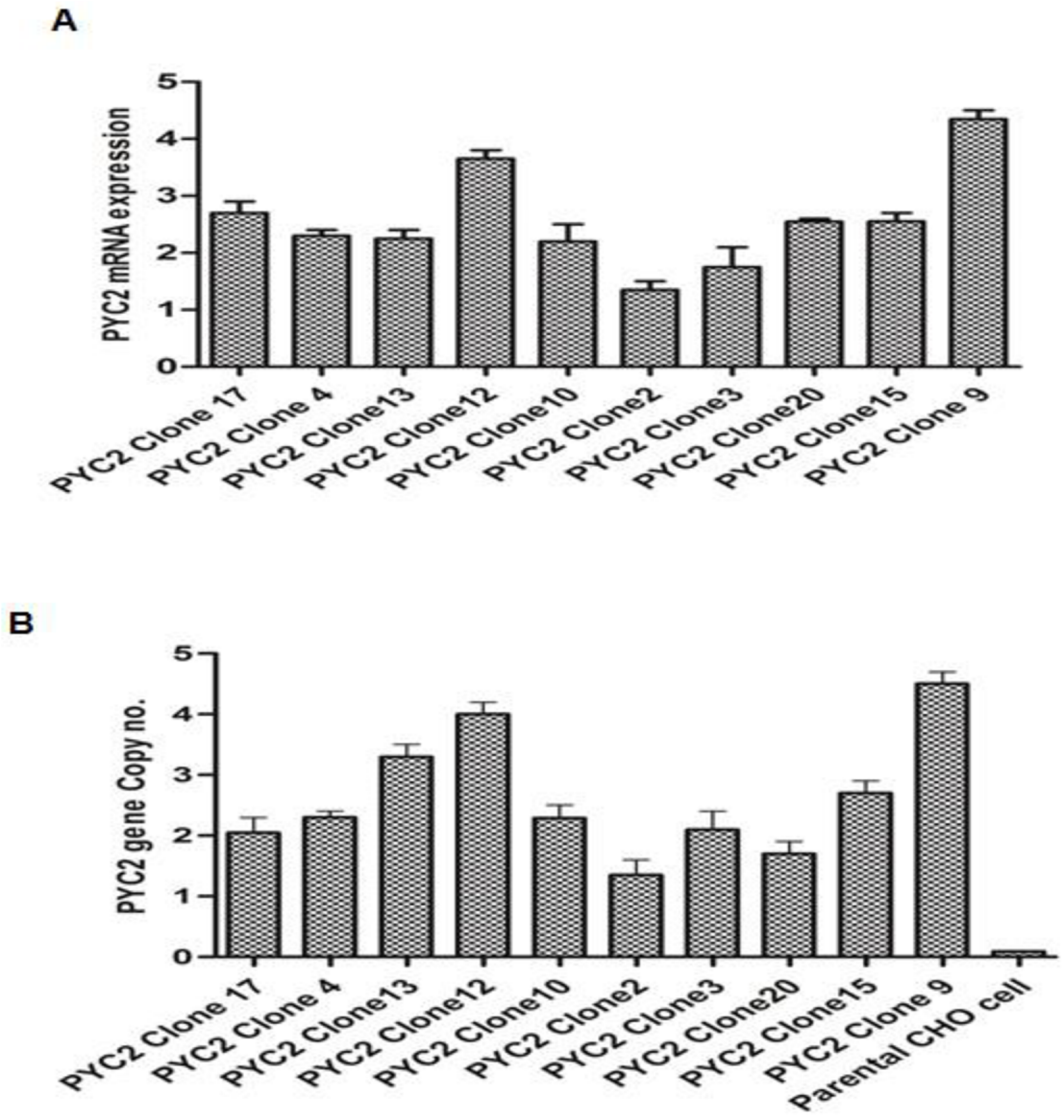
Gene copy number and mRNA expression analysis of the PYC2 expressing clones. **(A)** Fold expression of the PYC2 m-RNA relative to the house keeping β-actin gene (cDNA used as a template). (**B)** Calculation of gene copies relative to house-keeping β-actin gene for selected PYC2 clone#12 (genomic DNA used as a template).

It was also interesting to observe that, the engineered cells have shown a different growth pattern (viability, cell density and metabolic profile) than exhibited by control parental CHO cells. The clones were screened and selected based on their culture performance as well as mRNA expression profile obtained in a fed batch shake flask process **(Fig 3A).** To understand the gene load and its effect on the metabolic behavior of the selected PYC2 clones, we performed the qPCR (Quantitative polymerase chain reaction) by applying a relative quantification method 2^- ΔΔCq^ with the assumption of 100% PCR efficiency. We observed that the gene load of the selected PYC2 clones varies in each clone and ranged from 2–4 copies **(Fig 3B).**

In CHO cells, the endogenous pyruvate carboxylase enzyme converts pyruvate into oxaloacetate, which then turns into malate. Finally, the synthesized malate transported into the mitochondrial matrix through the malate-aspartate shunt. Expression pattern of the endogenous pyruvate carboxylase is not sufficient to efficiently drive the accumulated cytosolic pyruvate into the mitochondrial metabolic networking system [28].

In PYC2 expressing clones, the functional copy number of the PYC2 gene played a pivotal role in the reduction of pyruvate concentration in the cytosol as well as in improving the cellular ΔL/ΔG ratios **(S1 Table).** In the shake flask fed-batch study, we observed that each clone have shown a significant variation in the glucose/lactate metabolism as well as cell viability profile. The first consequence which can be inferred from over expressing PYC2 gene depends on the presence of the functional PYC2 gene load in the CHO genome.

In this particular set of selected clones, only clone#12 has shown an increased cell density and high lactate consumption till stationary phase compared to other clones. Based on overall culture performance and PYC2 gene copy load, we selected clones#12 amongst top 10 clones and further analyzed their phenotypic, metabolic behavior as well as amino acid profile in a fed-batch mode from the exponential phase to the stationary phase of the cell culture process.

### Phenotypic and metabolic study of PYC2 modulated clone

#### Cell growth profile (cell density and viability)

**Fig 4:**
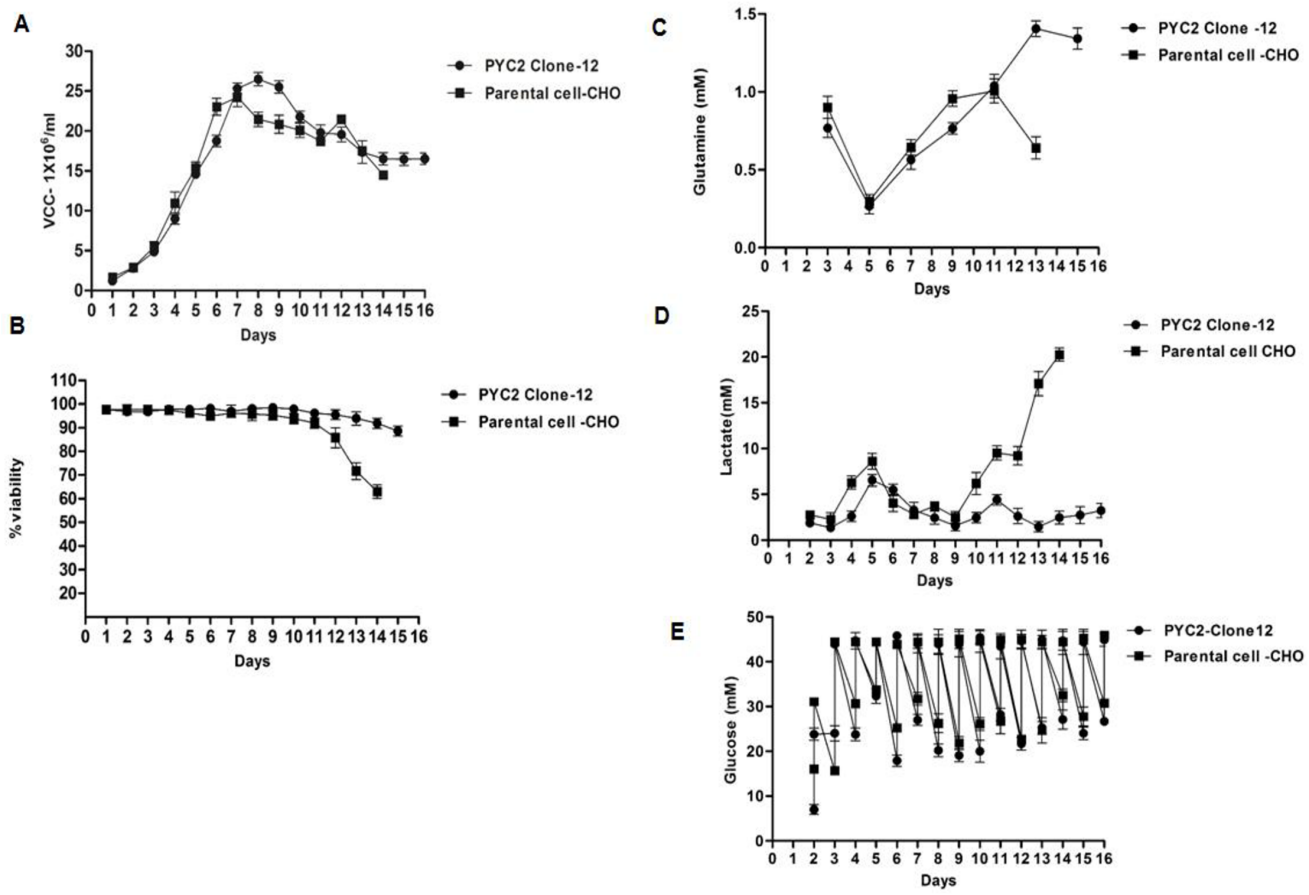
Culture performance of the clone#12 expressing PYC2 and parental CHO cell in shake flask fed-batch culture. **Graph representing culture profile: (A)** Cell density,(**B)** cell viability, (**C)** Glutamine (**D)** lactate and (**E)** glucose consumption profiles.

As mentioned above, based on the culture performance, we selected the PYC2 clone#12 for comprehensive study of the metabolic profile of the cells. We performed a comparative shake flask fed-batch study of the PYC2 expressing clone (#12) and parental CHO cells for their phenotypic and metabolic behavior in an optimized cell culture condition. The parental cells and PYC2 clone were cultivated to grow in a basal medium CD-Forti-CHO supplemented with 6mM glutamine in appropriate cell culture conditions. In the fed-batch cell culture study, the Acti-feed A and B were supplemented and fed the culture from day 3 onwards till one day prior to the batch harvest. To understand the cell’s central carbon metabolism, we supplemented the culture with high glucose 8g/L (44.5mM) feed from day 3 onwards to the batch ends **(Fig 4E)**. The shake flask data depicts that the PYC2 expressing clone (clone#12) and parental cells showed a stagnant exponential growth with the cell density of approximately 26 million/ml and 20 million/mL, respectively, which was nearly 6 million/mL higher cell density in clone #12 compared to the parental CHO cells **(Fig 4A)**. We also observed an extended cell viability in the PYC2 clone which was more than 94.3% till day 14, however, a sharp viability drop was observed in case of parental CHO cells and went down up to 64% on day 14 **(Fig 4B)** In earlier study carried out with the PYC2 expressing CHO cells, the maximum cell density reported so far, falling in the range of 10–14 million cells/mL which is around 10–12 million cells/mL lesser as compared to the current achieved in PYC2 clone#12. The viability profile of PYC2 expressing clone#12 is better until day 14, however, in previously published report viability of the PYC2 expressing clone reported maximum up to 75% till day 12 [26,28,33,35].

#### Cell energy metabolism (NAD+/NADH flux)

Over-expression of PYC in cells would have a higher input of reducing power from the cytosol into the mitochondria due to the higher flux through the Malate-Aspartate shuttle, therefore increasing the cell’s energy metabolism, which could be explained by culture’s extended lifespan. We also studied the central carbon metabolism in the selected clone and observed that the NAD^+^/NADH ratio was significantly lower in clone#12 (0.17) as compared to the parental CHO cells (5.05) which showed an enhanced energy metabolism **(Table 1)** of the PYC2 engineered clone compared to the parental cells.

**Table 1:** Comparative NAD^+^/NADH ratios of parental CHO cell and PYC2 clone#12 in feed batch culture studies.

Cell Type	NAD+/NADH ratio
Parental CHO cells	5.05
PYC2 Clone#12	0.17

#### Cell metabolic profile (specific rate of glucose, lactate, and glutamine)

In addition to the cell analysis, we also evaluated the specific consumption and production rates of the metabolites in a fed-batch culture summarized in **Table 2**. In fed-batch culture, the average specific glucose consumption rate was 1.21±0.55 for the PYC2 clone where as 0.73±0.28 for parental cell line. In spite of high glucose consumption rate, a marked difference about 3 folds was noted with respect to the specific production rate of the lactic acid in PYC2 clone (0.15±0.02) as compared to the parental cell line (0.54±0.27).

**Table 2:** Specific consumption/production rates of metabolites in Parental CHO cell and clone #12 studied in shake flask fed-batch culture.

Specific Productivity	PYC2 Clone#12	Parental CHO Cells
**q**_**Glucose**_ (pmol/cell.day)	1.21±0.55	0.73±0.28
**q**_**Lactate**_ (pmol/cell.day)	0.15±0.02	0.54±0.27
**q**_**Ammonia**_ (pmol/cell.day)	0.22±0.14	0.31±0.16
**q**_**Glutamine**_ (pmol/cell.day)	0.04±0.06	0.06±0.04

To evaluate the metabolic state of the clone#12 and its efficiency towards central carbon metabolism, the stoichiometric ratio between lactate production and glucose consumption (ΔL/ΔG) was also studied. The lactate consumption rate of clone#12 was approximately 4 fold higher as compared to the parental cells **(Fig 4D).** Out of top 10 PYC2 expressing clones, few clones showed lower ΔL/ΔG ratio during exponential growth phase, however, it rose when culture entered into the stationary phase. Among the selected clones, the ΔL/ΔG ratio was stable only for clone #12 and found 0.029 and 0.016 at exponential and stationary phase of the cell cycle, respectively **(S1 Table)**. These finding indicates that the PYC2 engineered clone#12 have more efficient central carbon metabolism than the one exhibited by other clones as well as parental CHO cells.

We also assessed the metabolic behavior of the cells in terms of glutamine consumption which is a quick energy source for the growing CHO culture and observed that the glutamine consumption rate of the clone#12 was slightly lesser as compared to the parental cells **(Fig 4C)**. This can be interpreted that the PYC2 expressing clone#12 sufficiently activated their metabolic machinery using pyruvate flux through the conversion into pyruvate-oxaloacetate which consequentially reduces the glutamine consumption.

#### PYC2 engineered clone’s behavior in different culture medium

**Fig 5:**
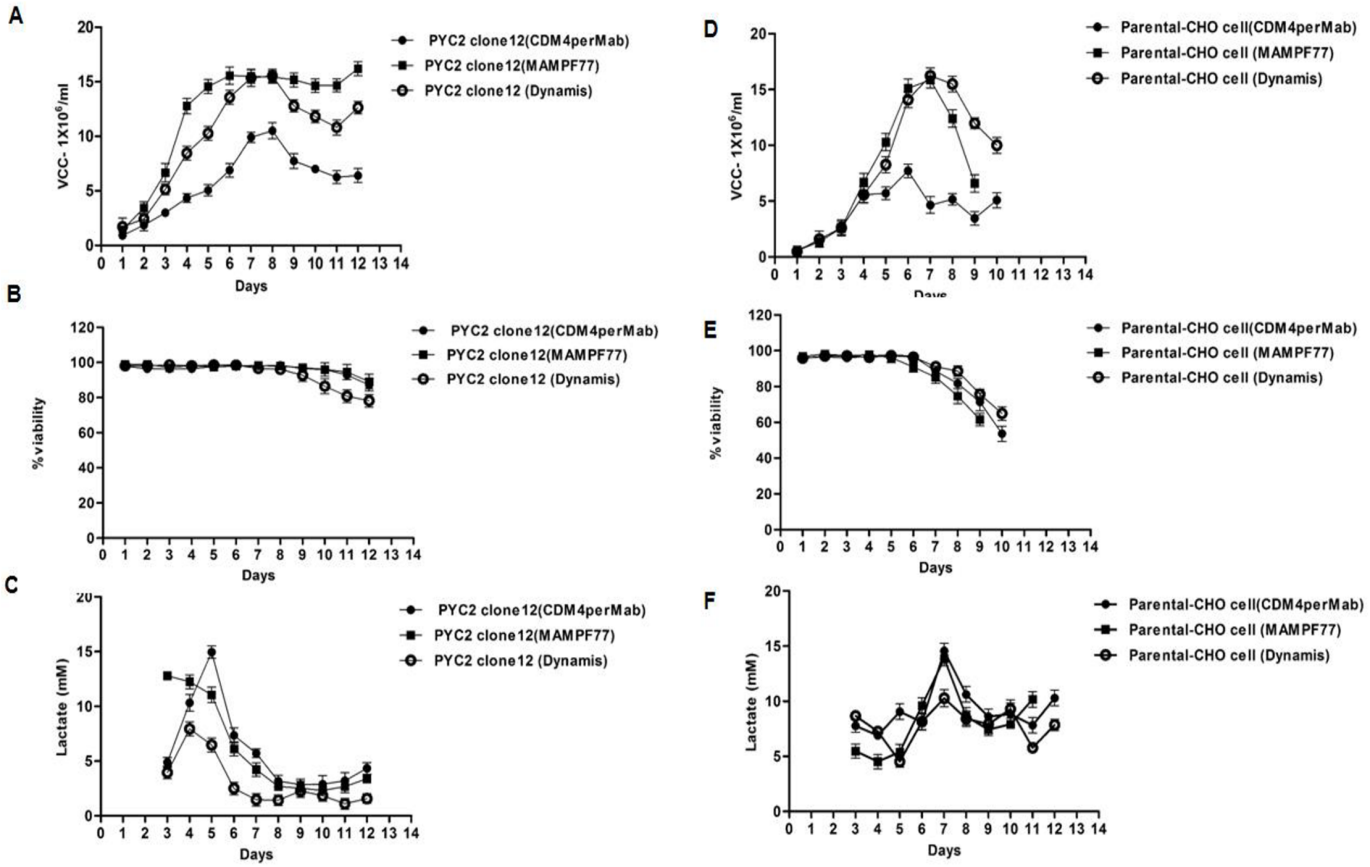
Culture performance of the clone 12 expressing PYC2 and parental CHO cell in culture medium MamPF77, CDM4perMAB and Dynamis in batch mode. Graph representing culture profile: (A and D) Cell density, (B and E) cell viability, (C and F) lactate profile.

To assess the metabolic behavior of the PYC2 engineered clone, subsequently, we also analyzed the clone#12 performance in other few routinely used commercially available chemically synthesized media. It was observed that, the cell viability of clone #12 in batch mode with basal media MamPF77 and CDM4perMab was significantly better than parental CHO cells. Culture was extended further greater days in all three media used for clone evaluation in comparison to the parental cell, however, there was no significant difference observed in terms of cell density in both PYC2 modulated clone as well as parental CHO cell (~ 15 million cells/mL) **(Fig 5A and 5D)**. However, the lactate accumulation found to be significantly slower and consumed faster in clone #12 during exponential phase of the growth as compared to the parental CHO cells in all three media **(Fig 5B, 5C, 5E and 5F)**.

To study the effect of PYC2 over expression on the cell metabolism, we evaluated above mentioned commercially available medium that are mostly used for the production of recombinant monoclonal antibodies. The idea was to study the effect of different components present in commercially available medium on the glucose and lactate metabolism of the cells. Earlier study reveals that, the amino acid composition and trace elements present in the cell culture medium affects the metabolic behavior of the cells, significantly. To see the effect of different medium, we cultured both parental as well as PYC2 modulated cells in different commercially available medium and studied the cell behavior and metabolic profiling of the same. Culture performance in terms of cell viability of the PYC2 clone#12 in batch mode with basal media MamPF77, CDM4perMab observed was better than parental CHO cell. In addition, the culture prolonged for greater days in all three media as compared to the parental cells. However, there is no significant difference in terms of cell density in the PYC2 modulated cells as compared to the parental CHO cell.

The lactate profile reveals that the consumption rate was significantly higher in clone #12 as compared to the parental cells. The current study suggest that PYC2 modulated cells are capable of growing and controlling the lactate accumulation better in three different commercially available medium as compared to the parental cells.

#### Amino acid metabolism

**Fig 6:**
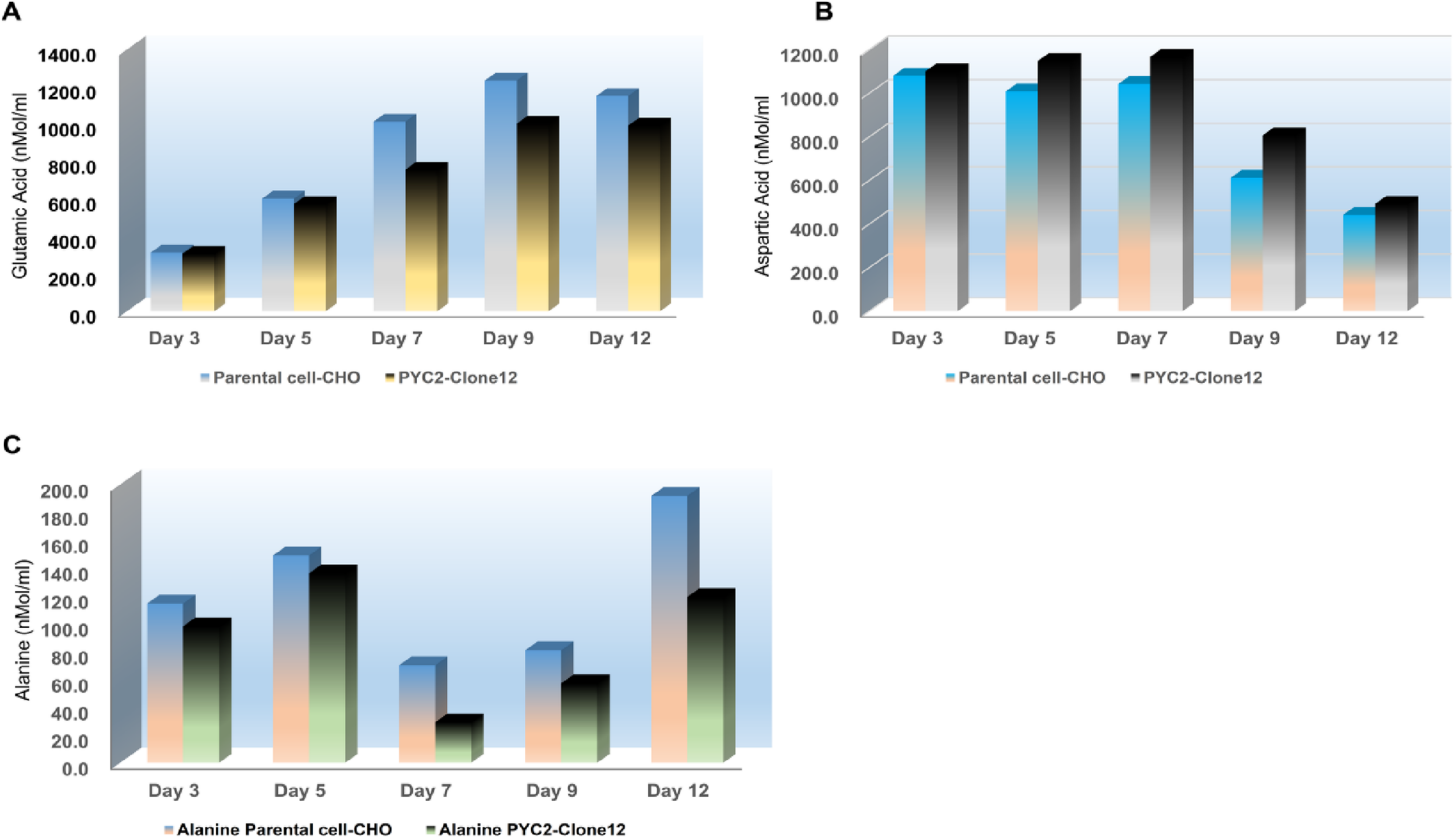
Amino acid analysis of spent medium. Concentration of the glutamic acid, aspartic acid and alanine profile of parental CHO cells and clone#12 grown in a fed -batch culture at different time interval. (**A**) Glutamic acid (**B)** Aspartic acid (**C)** Alanine.

Furthermore, we also focused on the analysis of the Amino acid synthesis and consumption profile of the clone#12 in comparison to the parental CHO cells **(S2 Table)**. Interestingly, unlike the parental cells, the bio-synthesis of the aspartate was enhanced and alanine concentration diminished by approximately two fold in PYC2 expressing clone#12 **(Fig 6A–6C)**.This can be explained by the effect of functional gene load of the PYC2 in the cytosol of the clone#12 which presumably increases the flux from pyruvate to oxaloacetate and eventually the increased oxaloacetate flux drives the bio-synthesis of the aspartate amino acid.

#### Antibody expression and its quality analysis

**Fig 7:**
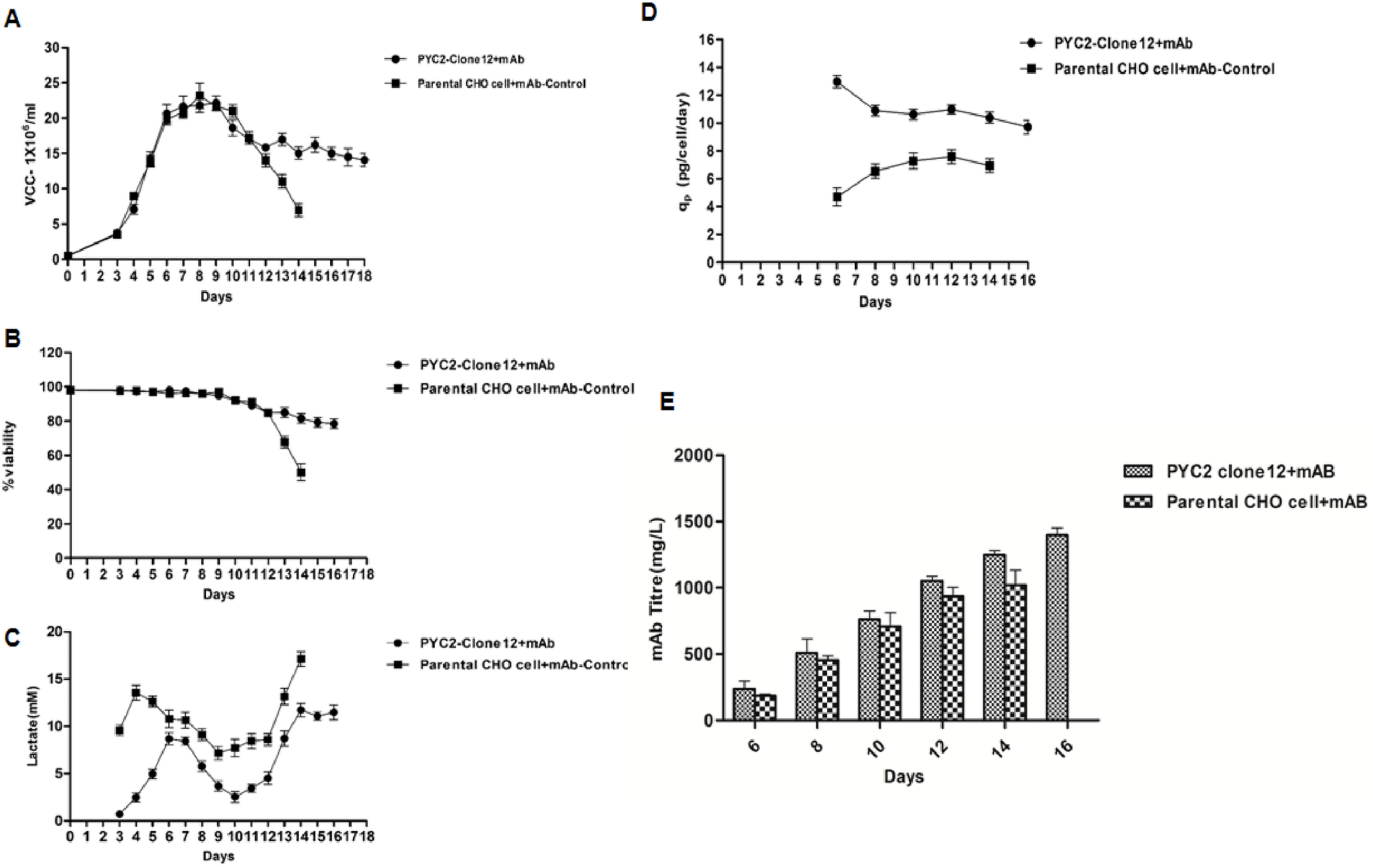
Comparative fed batch study of mAb expression in CHO-PYC2 clone 12 and CHO-S control cells. **(A)** Cell density, (**B)** Cell viability, (**C)** Lactate profile (**D)** Specific productivity and **(E)** Titer profile.

To assess the performance of PYC modulated cell line, the PYC2 clone (Clone#12) and parental cells were transfected with the expression construct harboring light and heavy chain gene encoding a therapeutic mAb. The cells were transfected using the same protocol as described earlier (electroporation) and subsequently stable pools were generated using various concentration of the selection agents. The stable pools generated from both PYC2 clone and parental cells were then studied in a fed-batch shake flask culture with the same strategies as done before. We analyzed the cell density, viability, glucose, lactic acid, protein expression and quality profiles. As expected, the CHO-PYC2 clone12+mAb (experimental) transfected cells showed extended cell viability up to 78% till day 18 as compared to the CHO+mAb cells (control) up to 40% till day 14 and 80% day 12. Early viability drop (below 80%) was observed in control CHO+mAb on day 13 as compared to the CHO-PYC2 clone12+mAb cells (experimental). However, the cell density profile was similar in both the cases **(Fig 7A and 7B)**. We also observed a mark reduction in the lactate profile compared to the control cells **(Fig 7C)**. A high glucose concentration up to 8g/L/day (44.5 mM) was maintained in both the culture throughout the experiment as explained earlier. The HPLC-Protein-A assay was used for the mAb’s titer assessment from the culture supernatant collected in different days. A significant increased in mAb titer up to ~70% higher (~1550mg/L, day 18) was obtained in PYC2+mAb culture (day18) as compared to the control CHO+mAb culture (903mg/L, day 14). The titer analysis of the experimental cells was done till day 18 as batch was extended till viability drops up to 78%, in contrast the titer profile of control cells was assessed up to day 14 as sudden viability drop was observed from day 13 onwards **(Fig 7B and 7D)**. The specific productivity (qP) of the experimental cells on day 18 was approx. 45% higher (6–7) compared to the control cells (4–5). The early cell viability drop and lesser protein titer in the control cells can be correlated with the lactate profile of both the cultures during exponential and stationary phase of the cell cycle. The lactate accumulation in the control cells was approximately 2 fold higher between day 12–13 (9-10mM) than that of the experimental cells (4-5mM) **(Fig 7C)**, which suggest that the PYC2 expressing cells (experimental) have shown better lactate consumption profile as compared to the control cells when mAb expression studies was performed in a fed batch culture mode. To date, this is a significant improvement reported in the mAb titer profile at cell pool stage and we expect further titer and cells performance improvement after clonal cell selection and upstream process optimization.

**Fig 8:**
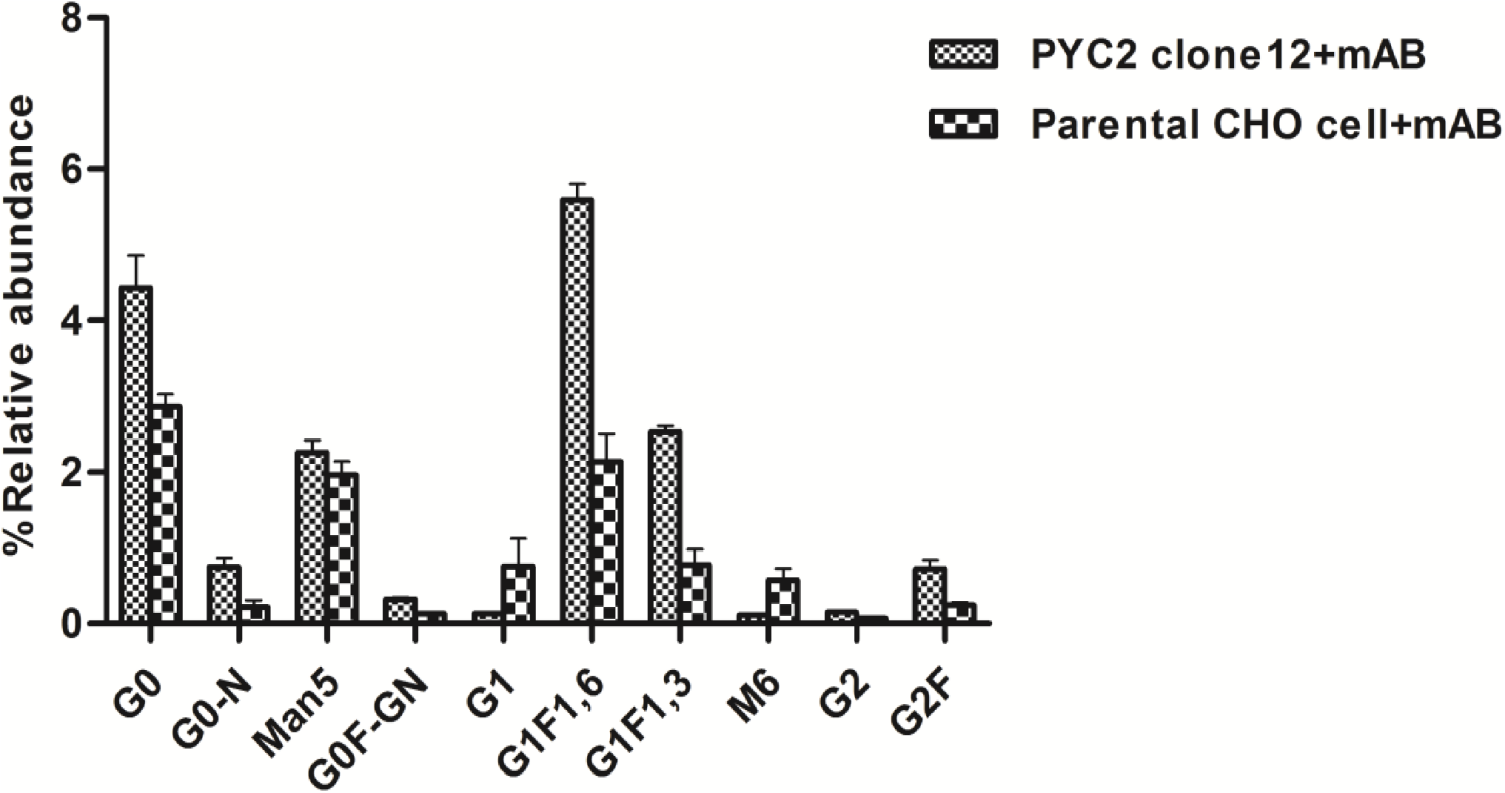
Comparative glycoform analysis of mAb secreted from the clone expressing PYC2 gene and a set of control without PYC2 over-expression. Relative abundance of glycan composition of a mAb.

To demonstrate the quality of the mAb protein, we collected the samples from the grown culture and analyzed the N-glycosylation patterns of the expressed protein. The glycosylation pattern of a mAb plays a pivotal role in CDC (complement-dependent cytotoxicity) and ADCC (Antibody dependent cell cytotoxicity) activity and significantly affects the efficacy of the molecule. Furthermore, we investigated the impact of PYC2 on various glycosylation patterns which is one of the critical quality attributes of an active and potential mAb. We observed a significant impact on the total glycosylation distribution of a mAb when expressed in both the expression hosts. The result obtained shows a total mannosylation reduction by 2 times and galactosylation improvement by 3 times as compared to the control cells **(Fig 8)**. Relative abundance of glycan pattern of G_0_F-GN, G1 and G2F were found comparatively 3–8% higher and a significant reduction of Man5/Man6 with PYC2 over-expression in mAb producing CHO cells.

It is well studied that different host cell can add different glycoforms to the monoclonal antibodies. The Fc glycan of human serum IgG predominantly contains G0F, G1F and G2F glycoforms and a trace amount of sialic acid. The recombinant antibodies produced in CHO cells exhibit similar glycoforms to the naturally occurring human serum IgG. The degree of sialic acid present in the FC-fusion proteins (I.e. Etanercept) affects the molecule’s potency but it doesn’t affect the IgG derived monoclonal antibodies [41,42].

The sialylation of mAb occurs at the terminal end of a glycan core structure, which is added after Gal addition. In the current study, glycosylation study was performed using HILIC-HPLC column, all glcyoforms of the mAb can be detected as shown in Fig 8, but, however, the sialic acid was not detected due to low or negligible abundance. The sialic acid level was below the detection limit (HPLC) in both control as well as experimental samples. We also studied the glycan composition of some of the commercially available mAbs and observed less than 1% sialic acid. The abundance of sialic acid also dependent on the host, clone and upstream process used for the monoclonal antibody development.

## Discussion

In the current study, we developed an engineered CHO cell line to control the glucose metabolism which exhibited higher cell density, extended lifespan and efficiently controlled central carbon metabolism. It is earlier studied that, the abundance of endogenous pyruvate carboxylase (PYC2) enzyme is quite low in CHO cells and a high rate of glycolysis of a cell culture process leads to high accumulation of pyruvate in the cytosol which is poorly transported to the mitochondrial metabolic network and resulting lactic acid accumulation in the cytosol [43].

Although, yeast pyruvate carboxylase gene has been engineered to tune up the cellular metabolic activity in various mammalian cell lines (CHO, BHK21and HEK2) which were used as a host for the expression of many recombinant products [9,29,44], however, the current study shows a superior over-all cellular performance of PYC2 engineered cell in terms of cell density, viability, lactate consumption, expression and product quality attributes. In engineered BHK21 cells, achieved high cell density, had an extended lifespan and also exhibited a more efficient metabolism producing less lactate per glucose consumed [28]. In another study done by Fogolin and collaborators, where in PYC2 was over -expressed in CHO cells showed drop in cell density, but at the same time cells exhibited improved lifespan and cellular metabolism [44,45]. In the current study, we used a codon optimized yeast pyruvate carboxylase gene for the enhanced gene expression in CHO cells for the re-wiring of the metabolic connectivity of the cytoplasm and mitochondria. To develop a metabolic efficient CHO cells from the PYC2 transfected heterogeneous pool, we applied a two-phase selection strategies and advanced sophisticated instruments to ensure an efficient engineering and selection of the modified cells without ambiguity on its clonality. The two-phase selection strategy allowed us to develop a cell line with increased gene copy load that has led to a better metabolic control during the cell culture process. Implementation of two-phase selection strategy also allowed elimination of low and poor producer clones when applied various concentration of selection agents (MTX and puromycin). In a fed-batch shake flask study carried out for cell pool selection, amongst the three pools, pool 2D (selected at 50ug/mL puromycin and 1000nM MTX) has shown relatively a better culture performance in terms of cell viability, longevity, cell density and lactate production as compared to other two pools. We presumed such behavior of the cells mainly due to selection of the PYC2 expressing cell pools exhibiting medium to high increased gene load. Subsequently, we used pool 2D cell population to screen a single cell which after screening and selection showed an optimized expression of PYC2, thus enhanced metabolic activity.

Traditionally, manual limiting dilution cloning method been commonly performed for the clone screening and selection, which is relatively labor intensive and time consuming which also doesn’t ensure the clonality. In our cell line engineering program, we used advanced sophisticated modern tools such as FACS for cell sorting and live cell imager to keep the track of colony generation and ensure cells clonality. This strategy allowed us to screen the potent clones from heterogeneous population of the PYC2 expressing pool.

In previous study, it is reported that the over expression of the yeast pyruvate carboxylase in CHO cell enhances the central carbon metabolism and exhibited maximum cell density up to 12–14 million cells/mL and viability up to 80% on day12. Where as in current study, we were able to achieve a cell density of up to 26 million/mL and extended cell viability up to 80% till day 16 as compared to the control cells which showed 12–14 million cells/mL and viability drop on day 12. This variation in metabolic profile, cell density and viability pattern of the selected clone attributed may be due to difference in the PYC2 gene load, integration site and cellular metabolism of each clone. As we know that the central carbon metabolism of each clone depends on the efficient utilization of pyruvate and conversion into ATP through TCA cycle [46]. Hence, variation in gene copy load of PYC2 gene may play a pivotal role on the central carbon metabolism of each clone. The PYC2 m-RNA expression studies done by RT-PCR reveals that, there is a drastic variation in the expression of the PYC2 gene in each clone and thus, reflected in the performance of the cell culture fed batch process. In fed batch study of the selected clones, reduction of *ΔL/ΔG* ratio is accompanied by variation in the expression levels of certain genes that participate in the central carbon metabolism and also showed that the existence of multiple steady states in culture with distinct cellular metabolism. Each clone showing a different metabolic state is represented by different lactate to glucose stoichiometric ratios of ΔL/ΔG. All together this work is able to compare the effect of PYC2 expression on the various parameters of the culture and evaluate their suitability for industrial use. Among the selected clones, clone#12 showed a maximum cell density up to 26 million cells/mL with viability of up to 90% till day 14 which is a very useful outcome for the biopharmaceutical production, if using an engineered host. We also demonstrate that, accumulation of metabolic wastes lactate and ammonia significantly reduced in clone#12 which in turn undoubtedly delays the acidification of the culture medium and may explain, at least in part, the higher maximum cell concentration and good product quality achieved. Noticeable higher cell density, extended viability and optimum ΔL/ΔG ratio of clone#12 indicates that it will prove as an excellent expression host for the production of therapeutic proteins in bio-pharmaceutical industries.

Further, when mAb protein was expressed in PYC2 engineered clone#12, a significant higher protein expression (up to 70%) was observed when batch was extended up to day 18. In contrast, the control CHO+mAb cells can sustain up to 12 days only with the same viability and shown 30% lesser protein expression on day 14 as compared to the experimental cells on the same day. The experimental cells have shown 70% higher expression since the batch was continued till day 18 which can be explained by may be due to a better lactate consumption profile in PYC2 engineered cells. Earlier studies revealed enhanced protein expression in PYC2 engineered cells maximum up to 25% [33] whereas the current study showed significantly higher protein expression of up to 70% at pool stage of the engineered cells. The expression may go even higher after clonal selection and upstream process optimization. The lactate accumulation in the control cells was 2 fold higher 9-10mM on day 12/13 as compared to the engineered cells (4-5mM). The lactate profile reveals a better lactate metabolism control exhibited by PYC2 modulated cell line.

In another part of the study, we explored the potential role of PYC2 expression on the quality of a bio-pharmaceutical product. The production of therapeutic antibody with a desiredglycoform profile has been and remain a challenging task in the bio-pharmaceutical industry [2]. Many successful attempts at modulating protein glycosylation toward desired patterns have been shown by media optimization and addition of various heavy metals [47–49]. In cellular system N-glycosylation patterns are known to be largely dependent on factors such as glycosylation processing enzymes, the availability of intracellular nucleotide sugar substrates and metabolic activity of the cellular system [50,41].

In this study, we demonstrated the impact of PYC2 over expression on titer and glycosylation pattern of a therapeutic monoclonal antibody produced in CHO cells.

The batch study was performed at the pool level with heterogeneous population of the CHO cells exhibiting variation in mAb expression. At the pool stage, we observed expression of the mAb protein up to 70%% higher compared to the control cell. A significant increase of mAb titer in the co-transfected pool could be because of two reasons, firstly the selected pool may possess increased PYC2 gene load which would have helped in controlling the glucose and lactate metabolism and secondly, the cells growing with higher cell density with control lactate would have allowed increased culture longevity.

In terms of quality of the product (mAb), we achieved three-fold increased total galactosyaltion composition of a therapeutic mAb when used PYC2 engineered cell line which in turn will reduce the extra efforts put in for cell culture process optimization for the glycosylation improvement. Previous studies have shown that the distribution of different glycoforms on the Fc region can significantly impact the mAb efficacy, stability, and its effector function [38]. In the process N-glycans synthesis, multiple sugar moieties can be added to form different glycoforms, e.g., G_0_, G_1_, G_2_, afucosylated complex, mannosylation. For recombinant mAb, glycosylation plays an important role for complement-dependent cytotoxicity (CDC) and antibody-dependent cell-mediated cytotoxicity (ADCC) functions through modulating the binding to the Fcγ receptor [37,40]. In recombinant mAb specific glycosylation pattern and binding to the Fcγ receptor is necessary to achieve therapeutic efficacy.It is well documented that glutamine, media condition, pH, lactate and dissolved oxygen (DO) had significant effects on the relative percentage of various glycans of mAb [42]. When we studies the impact of increased PYC2 co-expression with mAb, interestingly we observed that the PYC2 not only enhances the mAb titer but also affected the overall glycosylation pattern. Terminal mannose (Man) residues that have high affinity with various types of receptors in human increases the clearance rate of the therapeutic protein [42]. Total mannosylation was two-fold lesser in PYC2 expressing cell which may help in enhancing the half-life of a mAb protein.

## Conclusions

In conclusion, the current study reveals that, an increased expression of the PYC2 gene in CHO cells enhances its metabolic activity, which in turn directly or indirectly affected the overall cells performance, including titer and glycosylation composition (pos-translational modification) of a therapeutic protein. We also demonstrated that the PYC2 engineering in CHO cells can be helpful in improving the titer as well as quality of a recombinant therapeutic protein potentially by tuning the CHO metabolic pathway. Since the lactate metabolism of the PYC2 engineered cell was tightly controlled as compared to the parental cells, the lactate metabolism attributed to a significant increased cell density and culture longevity in a fed-batch culture. The culture longevity and high viability have led to enhanced specific productivity of a mAb as compared to the control cell. In complex cellular metabolic network, our results suggest that, by enhanced expression of PYC2 gene, it is also possible to improve the culture performance with appropriate cellular redox condition. This work is able to compare the effect of PYC2 expression on the various parameters of the cell culture and evaluate their suitability for industrial use. Further, PYC2 engineered clone that has shown better performance amongst all can be used as an expression platform for cell line development and well controlled upstream process in bioreactor for the production of an affordable therapeutic recombinant protein for a large population.

## Materials and methods

### Cell line, medium and culture conditions

The suspension CHO-S cell line (Thermo Fisher Scientific, USA) is used as a host cell. We used CD-Forti CHO growth medium (Thermo Fisher Scientific, /Gibco, USA) for cloning, screening and batch studies. The cell growth medium is supplemented with 6–8 mM L-glutamine (Thermo Fisher Scientific/Gibco, USA), 1X anti-clumping agent (Thermo Fisher Scientific, USA) and different concentration of the selection agent Puromycin (Thermo Fisher Scientific, USA), and Methotrexate (MTX) (Sigma Aldrich, USA). The culture was grown in a CO_2_ incubator set at 37°C, 85% humidity, CO_2_ 8% Growth medium MAMPF77 (Bioconcept AMIMED, Switzerland), CDM4MAB (Hyclone-GE,USA-UK) Dynamis (Invitrogen, USA) used as per manufacturer’s instruction_._

### Gene and oligonucleotide synthesis

The gene encoding yeast cytosolic pyruvate carboxylase (PYC2) was chemically synthesized and codon optimized for CHO cells was received from GeneArt, Germany (now part of Life Technologies/Thermo Fischer Scientific, USA). The codons were optimized with an average GC content of 62% to achieve an increased expression of enzyme pyruvate carboxylase. Codon adaptation index (CAI) of the optimized sequence is 0.97.The codon optimized synthetic gene is cloned into an appropriate mammalian expression vector for the CHO transfection, propagation and subsequently clone selection. The DNA sequence of sub-cloned gene was verified by DNA sequencing and found intact. Oligonucleotides used for gene integration, DNA sequencing and RT-PCR (real time PCR) studies were designed in-house and procured from Sigma Aldrich, USA.

### Vector construction

The PYC2 gene bearing plasmid pMPYC-CHO was constructed (vector backbone used from Invitrogen/Thermo Fischer Scientific, USA) using CHO cell codon optimized synthetic pyruvate carboxylase gene derived from the yeast *Saccharomyces cereisiae*. The pMPYC vector possesses kanamycin and puromycin antibiotic resistance markers for clone selection in *E*.*coli* and CHO cells, respectively. In addition, the expression plasmid also contains another selection marker DHFR (Di-hydrofolate reductase) which was used as dual selection marker for the stringent phenotypic selection of stably transfected cells in the presence of varying concentration of MTX. Codon optimized PYC2 gene fragment was cloned at Avr*II* and BstZ*II71* cloning sites (MCS) of the pMPYC vector. The *E*.*coli* strain DH5-alfa was transformed with the ligated product for the colony formation as well as *E*.*coli* clone selection in presence of 30ug/mL kanamycin. The DNA sequences of cloned genes were verified by DNA sequencing before CHO transfection and cell line engineering.

### Transfection of CHO cells

CHO cells were transfected with the pMPYC construct encoding the gene yeast pyruvate carboxylase using Neon electroporator (Neon Transfection System, Invitrogen/Thermo Fischer Scientific, USA). Prior to transfection, the plasmid DNA was isolated and purified in bulk and further linearized with restriction enzyme Nru*I*.

A day before transfection, the CHO-S cells were cultured at 1.0× 10^6^ cells/mL with more than 95% viability in a complete CD-Forti-CHO medium and incubated in a shaker at 140rpm, 37°C, 80% relative humidity, and 8% CO_2_ for about 24 hours. On the day of transfection, cells were analyzed for high viability and harvested the required number of cells by centrifugation at 200 × g for 5 minutes. The cells were cultured at 4.0 × 10^7^ viable cells/10mL in a T75 flask for 25μg plasmid transfection. We electroporated the CHO-S cells with the linear pMPYC plasmid at 1500V for 10ms and 3 pulses. The electroporated cells were then transferred to a T75 flask containing 10ml CD- Forti-CHO and 8mM glutamine and immediately incubated at 37°C, 80% relative humidity, and 8% CO_2_ for stable integration. To develop a cell line that produces optimized expression of the PYC2 enzyme, we applied a two-phase selection scheme with dual selection marker that has generated four stable pools of stably-transfected cells confirming the gene integration into the CHO genome. We performed the pool selection using a complete CD-Forti-CHO medium using various combinations of selection agent puromycin and MTX. Subsequently, we performed single cell cloning using FACS (Fluorescence activated cell sorting) (BD bioscience, USA) a cell sorter in which live cell population were gated using forward and side scattered (FSC and SSC) as function of integral area, height, width and granularity.

### Cell pool generation and clone selection

Two days post-transfection, the cell pools were subjected to puromycin and MTX selection with 10-20ug/mL and 100-200nM (Phase-I), respectively. Subsequently, the phase I selected pools were subjected further to higher concentration of puromycin and MTX with 30-50ug/mL and 500-1000nM MTX (Phase-II selection), respectively. The phase-II selected pool (2D) was further cloned by single cell sorting into 2x384 well plates with the help of FACS cell sorter under white background (without using any labeled antibody). The plates were incubated in a static condition at 37°C, 80% relative humidity, and 8% CO_2_ for about two weeks to allow the cells to grow and form the colonies. Each well of 384 plates was monitored to ensure colony formation from a single cell using the cell imaging system (Thermo Fischer Scientific, USA). The artifact, duplet and triplet colonies were disqualified and selected 40 colonies generated from single cell only. Further, the colonies derived from a single cell were expanded and screened for top10 clone selection based on PYC2 expression and cells growth pattern.

### PYC2 expression confirmation by RT-PCR

Total RNA from top 10 selected PYC2-CHO clones including CHO-S cells (parental cell) as a control was extracted using the Qiagen RNA extraction kit and the manufacturer's protocol. RNA concentration was determined by using nano-drop spectrophotometer, and its quality and integrity were checked by electrophoresis on 1.5% (w/v) agarose gel. The RNA was treated with DNase*I* (Thermo Fisher Scientific, USA) prior to cDNA synthesis as per manufacturer’s recommended. For reverse transcription (RT), we used 500ng of total RNA transcribed into cDNA in a 20μl reaction mixture performed using Qiagen reverse transcription kit and the oligo (dT)15 primers provided in the same kit. To check the PYC mRNA expression, gene-specific forward and reverse oligonucleotides were used (synthesized from Sigma Aldrich, USA) in a 20μl reaction mixture and carried out PCR for 40 cycles [Initially 95°C for 7min and cycle repeated for 95°C-15 second and 60°C (annealing and extension) for 30 seconds (Pikoreal, Thermo fisher Scientific, USA). Quantification was carried out using formula relative quantification method 2^- ΔΔCq^[51]. The PCR product was then electrophoresed on 1% (w/v) agarose gel to confirm the PYC2 expression of top 10 selected clones.

### Fed-batch shake flask study (pools and clones)

For a fed batch shake flask study, the selected cell pools and top 10 clones expressing PYC2 along with parental CHO cells were seeded with 0.5 × 10^6^ cells/mL into 30ml CD-Forti-CHO growth medium (Invitrogen/Thermo Fisher Scientific, USA) supplemented with 6mM glutamine and 50ug/ml dextran sulphate in a 125ml shake flask (Corning, USA) and incubated at 37°C, 8% CO_2_ at constant agitation of 140rpm in a CO_2_ shaker incubator (Kuhner, AG). In this study, we used Acti-feed A and B for fed batch supplement of shake flask culture. Feed supplementation was started from day 3 onwards and maintained according to manufacturer’s instruction. A high concentration 8g/L (44.5mM) of glucose was maintained through day 3 to batch end.

### Gene copy number analysis

To quantify the gene copy number of the PYC2 gene integrated into CHO genome, qPCR (quantitative PCR) was performed from genomic DNA of selected clones using Piko-real PCR system (Thermo Fischer Scientific, USA). The genomic DNA was isolated using Qiagen kit and manufacturer’s protocol. We used β-actin house keeping primers as an internal control and PYC2 gene specific primers to perform quantitative PCR **(S3 Table)**. Each reaction mixture contained 1X CYBER green dye master mix (Thermo Fischer Scientific, USA) and 20 pmole each set of primer. The qPCR experiment was performed as a 2 step protocol, initial denaturation step at 95°C for 7 minute and 60°C for 45 seconds followed by 40 cycles. We estimated the PYC2 gene copy number using relative quantification method 2^- ΔΔCq^ [34].

### Analytical methods

We determined the cell count and viability of shake flask cultures using Vi-cell counter (Beckman Coulter Inc., Fullerton, CA). The glucose, lactate, glutamine and ammonium concentration was determined using YSI biochemical analyzer-Model 2700 analyzer (Yellow Springs Instruments, USA). We used a multi-sampler vapor pressure osmometer (Wescor Inc., USA) to assess the osmolality of the shake flask culture. To quantify the NAD^+^/NADH ratios in parental CHO cells and engineered PYC2 expressing clones, we used NAD^+^/NADH Assay Colorimetric Kit (Abcam, USA) and followed the same protocol as provided by the supplier. The IgG concentration was measured using high-performance liquid chromatography (HPLC, Waters, USA) Protein-A affinity column (Analytical column). The protein samples were collected at different time intervals to analyze the antibody titer.

### Glycan assessment by HILIC BEH amide column

The Acquity UPLC H class (Waters, USA) system was used for antibody Glycan analysis for the culture supernatant generated from the shake flask experiment. An Acquity UPLC BEH Glycan chromatography column (1.7um, 2.1mm x 150mm) was used with a flow rate 0.5 to 0.25 mL/min, column temperature 60°C, and sample storage temperature 2–8°C. The eluent mobile phase-A (50mM ammonium formate, pH 4.5) and mobile phase-B (Acetonitrile) were used to run the HPLC for glycan analysis. Detection was done using Fluorescence detector (λ ex = 330nm & λ em = 420nm). For sample preparation, Protein A purified sample 20μl each were denatured followed by deglycosylated using PNGaseF enzyme (NEB,USA) and incubated at 37°C for 3 hours. The deglycosylated samples were dried by speed vacuum concentrator (Thermo Fisher Scientific, USA) and then released N-Linked Glycans were labeled with 2-AB reagent (Waters, USA) by incubating in a heating block at 65°C for around 2.5 hours. Free 2-AB dye was removed by acetone clean-up followed by evaporation by dry heat. The dried sample was reconstituted with 20μL of Mobile Phase A and 30μL of Mobile Phase B buffers and injected 10uL on HILIC-UPLC BEH Glycan column for the separation of N- linked glycans. The peaks were identified by comparing with control samples and integrated for area % determination using Waters Empower 3.0 software.

### Amino acid analysis from spent medium

The Waters Acquity UPLC H class system was used for the spent medium amino acid estimation from the cell culture supernatant. The chromatography column AccQ.Tag Ultra C18 (1.7um, 2.1x 100mm, Waters, USA) was used with a flow rate of 0.7mL/minute, column temperature 43°C, sample storage temperature 2–8°C and UV detection at 260nm. The eluent mobile phase A (100% Waters AccQ.Tag Ultra Eluent A, Waters, USA), mobile phase B (10% Waters AccQ.Tag Ultra Eluent B in Milli-Q water, Waters, USA) mobile Phase C (Milli-Q water). For spent medium analysis, the harvest sample was filtered through 0.22μm syringe filter. Then took 10μL of sample/ amino acid standard (Waters, USA), 70μL of Borate buffer and 20μL of Derivatization reagent (Waters, USA) were mixed and heated at 55°C for 15 minutes. 1μl each sample and standard were injected on the above mentioned column. The amino acid standard used ranged from 10 to 250 pM for the quantitation of unknown amino acids. The Tryptophan is not available with Waters kit, therefore, tryptophan amino acid standard was prepared separately and the concentration of 100 to 1000nM was used for the estimation of unknown Tryptophan present in the cell culture media.

## Supporting information

S1 Fig Schematic flow diagram showing the cellular mechanism of central carbon metabolism. (DOC)

S1 Table Comparison of ΔL/ΔG ratio of the PYC2 expressing clones (selected clones) in fed- batch mode at exponential and stationary phase of the culture. (DOC)

S2 Table Concentration of Amino acid (nMol/ml) profile of the PYC2 clone #12 and parental cells with the function of time in a fed batch culture. (DOC)

S3 Table Oligonucleotide used for gene copy number analysis. (DOC)
