# Supplementary material for: Metabolic engineering of CHO cells for the development of a robust protein production platform
Source: PLoS One. 2017 Aug 1;12(8):e0181455. doi: 10.1371/journal.pone.0181455 (PMC5538670; doi:10.1371/journal.pone.0181455)
Supplement: S1 Fig — (DOC) [file pone.0181455.s001.doc]

**Supplementary information**

**S1 Fig.**

**
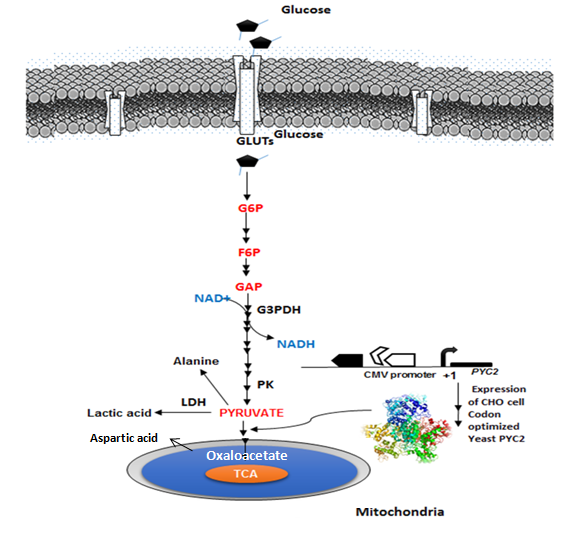
**

**S1 Fig. Schematic flow diagram showing the cellular mechanism of central carbon metabolism**.
