# Supplementary material for: Metabolic engineering of CHO cells for the development of a robust protein production platform
Source: PLoS One. 2017 Aug 1;12(8):e0181455. doi: 10.1371/journal.pone.0181455 (PMC5538670; doi:10.1371/journal.pone.0181455)
Supplement: S1 Table — (DOC) [file pone.0181455.s002.doc]

**Supplementary information**

**S1 Table.** Comparison of ΔL/ΔG ratio of the PYC2 expressing clones (selected clones) in fed- batch mode at exponential and stationary phase of the culture***.***

| **Name of the clone** | **Exponential Phase** | **Stationary Phase** |
| --- | --- | --- |
|  | ***ΔL/ΔG*** | ***ΔL/ΔG*** |
| **PYC2 clone 2** | 0.041 | 0.205 |
| **PYC2 clone17** | 0.072 | 0.0419 |
| **PYC2 clone 12** | 0.029 | 0.010 |
| **PYC2 clone10** | -0.025 | 0.075 |
| **PYC2 clone3** | -0.015 | 0.061 |
| **PYC2 clone15** | -0.067 | 0.132 |
| **Parental CHO cells** | 0.092 | 0.371 |
