# Supplementary material for: Metabolic engineering of CHO cells for the development of a robust protein production platform
Source: PLoS One. 2017 Aug 1;12(8):e0181455. doi: 10.1371/journal.pone.0181455 (PMC5538670; doi:10.1371/journal.pone.0181455)
Supplement: S2 Table — (DOC) [file pone.0181455.s003.doc]

**Supplementary information**

**S2 Table.** Concentration of Amino Acid (**nMol/ml)** profile of the PYC2 clone #12 and parental cells with the function of time in a fed batch culture:

| **Amino acid** | **Clone/Cell ID** | **Day 3-**  **conc.**  **nMol/ml** | **Day 5**  **conc.**  **nMol/ml** | **Day 7**  **conc.**  **nMol/ml** | **Day 9**  **conc.**  **nMol/ml** | **Day 12**  **conc.**  **nMol/ml** |
| --- | --- | --- | --- | --- | --- | --- |
| **Histidine** | Parental cell-CHO | 42.2 | 25.8 | 33.8 | 43.3 | 63.8 |
|  | PYC2-Clone12 | 30.6 | 39.3 | 23.5 | 43.7 | 43.5 |
| **Serine** | Parental cell-CHO | 272.4 | 166.5 | 142.9 | 64.5 | 130.6 |
|  | PYC2-Clone12 | 274.5 | 206.6 | 110.2 | 136.4 | 134.1 |
| **Arginine** | Parental cell-CHO | 59.7 | 45.6 | 27.1 | 23.0 | 105.5 |
|  | PYC2-Clone12 | 45.2 | 70.2 | 53.9 | 41.2 | 39.5 |
| **Glycine** | Parental cell-CHO | 61.5 | 72.2 | 165.5 | 210.2 | 224.0 |
|  | PYC2-Clone12 | 47.1 | 111.8 | 147.2 | 283.2 | 253.7 |
| **Aspartic acid** | Parental cell-CHO | 1080.9 | 1108.6 | 1042.5 | 641.8 | 462.6 |
|  | PYC2-Clone12 | 1100.4 | 1148.7 | 1167.7 | 807.6 | 493.2 |
| **Glutamic acid** | Parental cell-CHO | 314.0 | 603.1 | 1014.9 | 1234.8 | 1151.7 |
|  | PYC2-Clone12 | 307.7 | 574.6 | 757.3 | 1004.4 | 993.6 |
| **Threonine** | Parental cell-CHO | 145.5 | 147.4 | 225.8 | 209.8 | 228.2 |
|  | PYC2-Clone12 | 146.5 | 174.7 | 170.1 | 239.5 | 211.5 |
| **Alanine** | Parental cell-CHO | 114.5 | 149.1 | 70.2 | 80.9 | 191.9 |
|  | PYC2-Clone12 | 98.1 | 136.3 | 29.2 | 57.2 | 119.2 |
| **Proline** | Parental cell-CHO | 142.7 | 107.5 | 134.9 | 108.5 | 122.1 |
|  | PYC2-Clone12 | 139.5 | 143.4 | 113.1 | 139.9 | 120.0 |
| **Cysteine** | Parental cell-CHO | 78.3 | 74.4 | 84.4 | 68.6 | 95.3 |
|  | PYC2-Clone12 | 73.6 | 67.6 | 50.8 | 64.3 | 29.1 |
| **Lysine** | Parental cell-CHO | 137.9 | 112.4 | 124.6 | 88.6 | 139.1 |
|  | PYC2-Clone12 | 164.5 | 168.2 | 119.3 | 149.6 | 156.9 |
| **Tyrosine** | Parental cell-CHO | 63.1 | 52.4 | 90.7 | 74.1 | 86.3 |
|  | PYC2-Clone12 | 63.3 | 56.3 | 46.3 | 69.9 | 58.8 |
| **Methionine** | Parental cell-CHO | 53.3 | 37.1 | 47.9 | 37.4 | 43.8 |
|  | PYC2-Clone12 | 51.1 | 51.6 | 36.0 | 44.5 | 35.7 |
| **Valine** | Parental cell-CHO | 177.0 | 167.3 | 224.8 | 162.9 | 207.7 |
|  | PYC2-Clone12 | 175.1 | 214.5 | 182.0 | 250.1 | 216.6 |
| **Isoleucine** | Parental cell-CHO | 162.1 | 120.8 | 125.8 | 72.1 | 89.9 |
|  | PYC2-Clone12 | 156.2 | 160.5 | 113.6 | 120.7 | 85.9 |
| **Leucine** | Parental cell-CHO | 256.7 | 183.2 | 157.1 | 64.5 | 106.6 |
|  | PYC2-Clone12 | 247.5 | 248.5 | 153.2 | 147.7 | 102.3 |
| **Phenylalanine** | Parental cell-CHO | 83.7 | 70.7 | 123.9 | 105.8 | 122.9 |
|  | PYC2-Clone12 | 76.3 | 95.8 | 87.5 | 123.0 | 98.8 |
