# Supplementary material for: Metabolic engineering of CHO cells for the development of a robust protein production platform
Source: PLoS One. 2017 Aug 1;12(8):e0181455. doi: 10.1371/journal.pone.0181455 (PMC5538670; doi:10.1371/journal.pone.0181455)
Supplement: S3 Table — (DOC) [file pone.0181455.s004.doc]

**Supplementary information**

**S3 Table.** Oligonucleotides used for gene copy number analysis.

| **Oligonucleotide** | **Description** | **Size** |
| --- | --- | --- |
| PYC-forward primer qPCR/RT-PCR | CTGGTGCCTAACATCCCTTT | 20bp |
| PYC-reverse primer qPCR/RT-PCR | CTTCACAAAGTGGTCGATGG | 20bp |
| Β-actin forward primer | AGCTGAGAGGGAAATTGTGCG | 21bp |
| Β-actin forward primer | GCAACGGAACCGCTCATT | 18bp |
